# Going on Eighteen

**Published:** 1902-06

**Authors:** 


### TEXAS MEDICAL JOURNAL.

AUSTIN, TEXAS.

A MONTHLY JOURNAL OF MEDICINE AND SURGERY.

EDITED AND PUBLISHED BY
F. E. DANIEL, M. D.

ASSOCIATE EDITOR:
WITTEN BOOTH RUSS, M. D.,
San Antonio, Texas.

Published Monthly at Austin, Texas. Subscription price $1.00 a year in advance.

Eastern Representative: John Guy Monihan, St. Paul Building, 220 Broadway, New York City.

Official organ of the State Association of Health Officers, the West Texas Medical Association, the Houston District Medical Association, the Austin District Medical Society, the Brazos Valley Medical Association, the Galveston County Medical Society, and several others.

GOING ON EIGHTEEN.—This number completes Volume XVII of the *Red Back*, and July will begin its eighteenth year. It has been a success from the beginning, and has a hold on its readers and its advertising patrons well earned and maintained by the course it has pursued from the beginning in upholding the standard of medical ethics, in citing to higher profesional life and character, and by fearlessly denouncing quackery and shenanegan in any shape, in high places as well as in low. It has the cleanest and best paying subscription list and the best and most discriminating advertising patronage of any journal—north, south, east or west. Its seventeen years of successful work is a guarantee of stability and permanency, and the fact that most of its patrons have stuck to it the greater part of the time,—many from Vol. I, No. 1,—furnishes a guarantee that it is a *paying medium* for advertising those things which a doctor needs and ought to have.

We make our bow now to our patrons and start in on our eighteenth round with renewed courage and determination to still distance all competitors and to furnish, as always heretofore, a live, spanking and vigorous monthly visitor.

